# Supplementary material for: No evidence for ongoing replication on ART in SIV-infected macaques
Source: Nat Commun. 2024 Jun 14;15:5093. doi: 10.1038/s41467-024-49369-9 (PMC11178840; doi:10.1038/s41467-024-49369-9)
Supplement: Supplementary file 1 — Supplementary Information [file 41467_2024_49369_MOESM1_ESM.pdf]

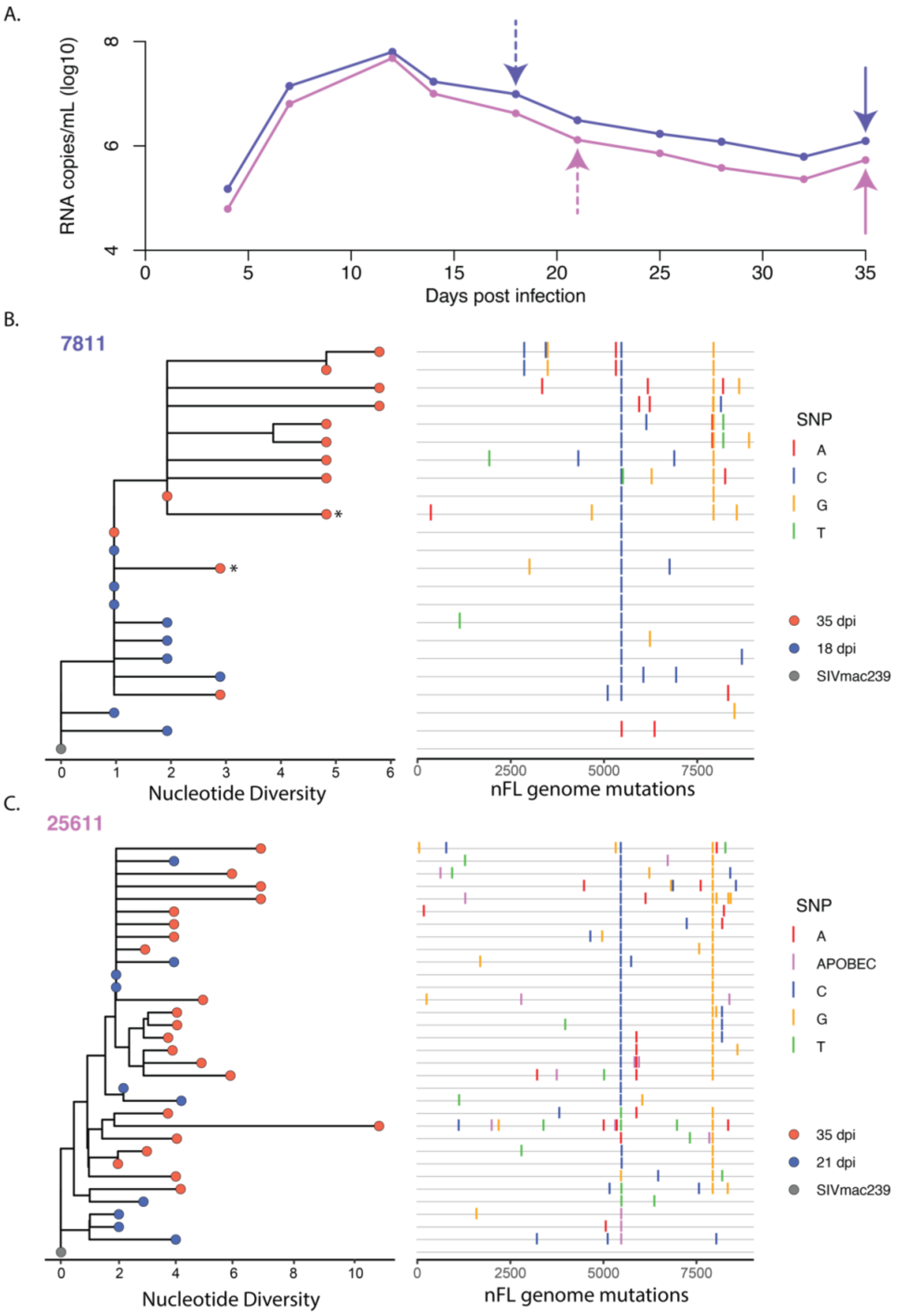

Supplemental Figure 1. Untreated, control RM plasma viral load and accumulated mutations. Plasma viral load curve with dashed and solid arrows indicate when baseline and a second time point sample were collected from each animal to perform viral RNA sequencing (A). Single nucleotide polymorphisms (SNP) from the SIVmac239 founder sequence [red for adenine (A), blue for cytosine (C), orange for guanine (G) and green for thymine (T), including potential APOBEC-mediated mutations (purple)] are indicated in the highlighter plot (B, C). Asterisks indicate sequences with short indels. Source data are provided as a Source Data file.

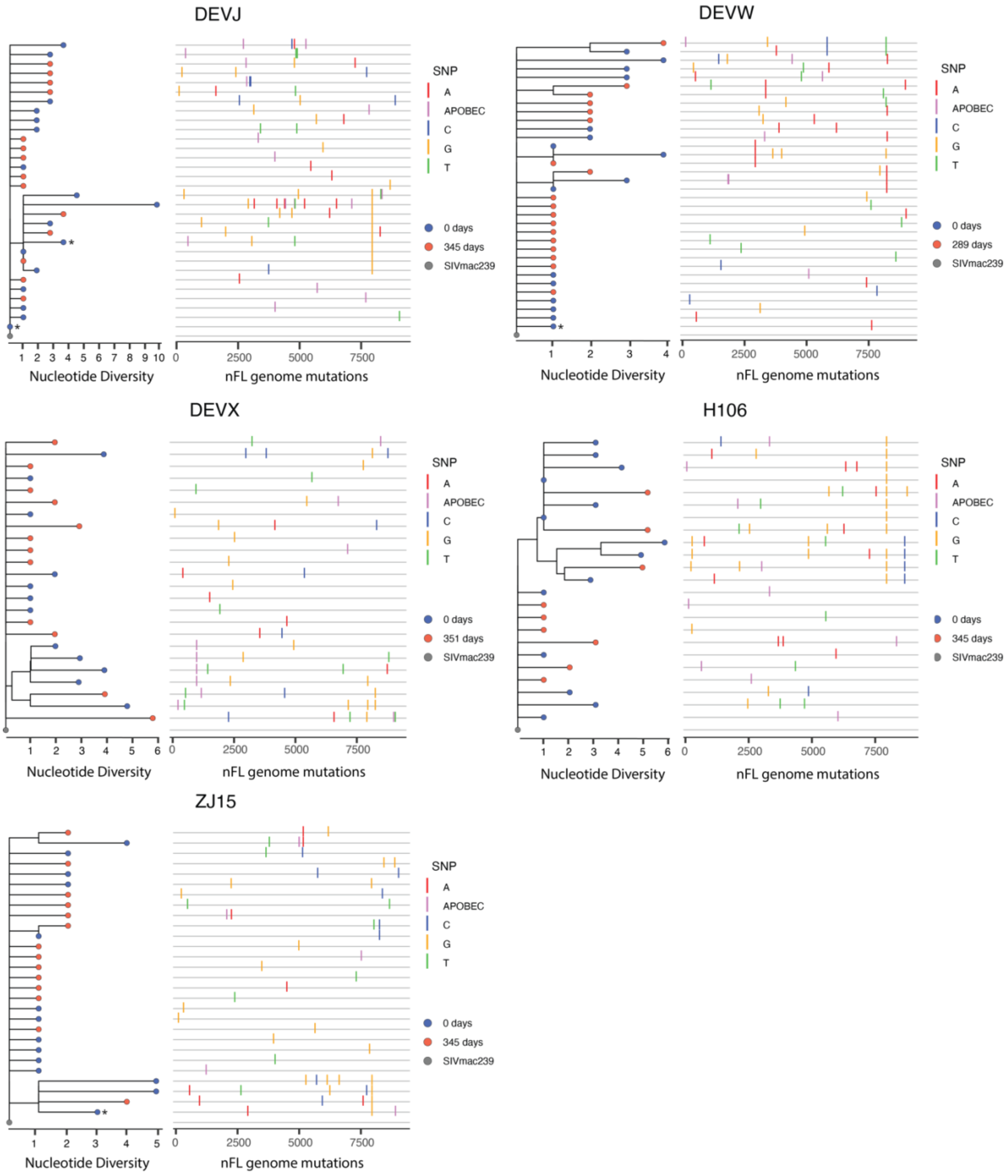

Supplemental Figure 2. Trees and highlighters for cohort 1 animals. Phylogenetic trees for each individual RM show early evolution pre-ART but not continued evolution after ART. Sequences with indels are indicated with dots. Single nucleotide polymorphisms (SNP) from the SIVmac239 founder sequence [red for adenine (A), blue for cytosine (C), orange for guanine (G) and green for thymine (T), including potential APOBEC-mediated mutations (purple)] are indicated in the highlighter plot.

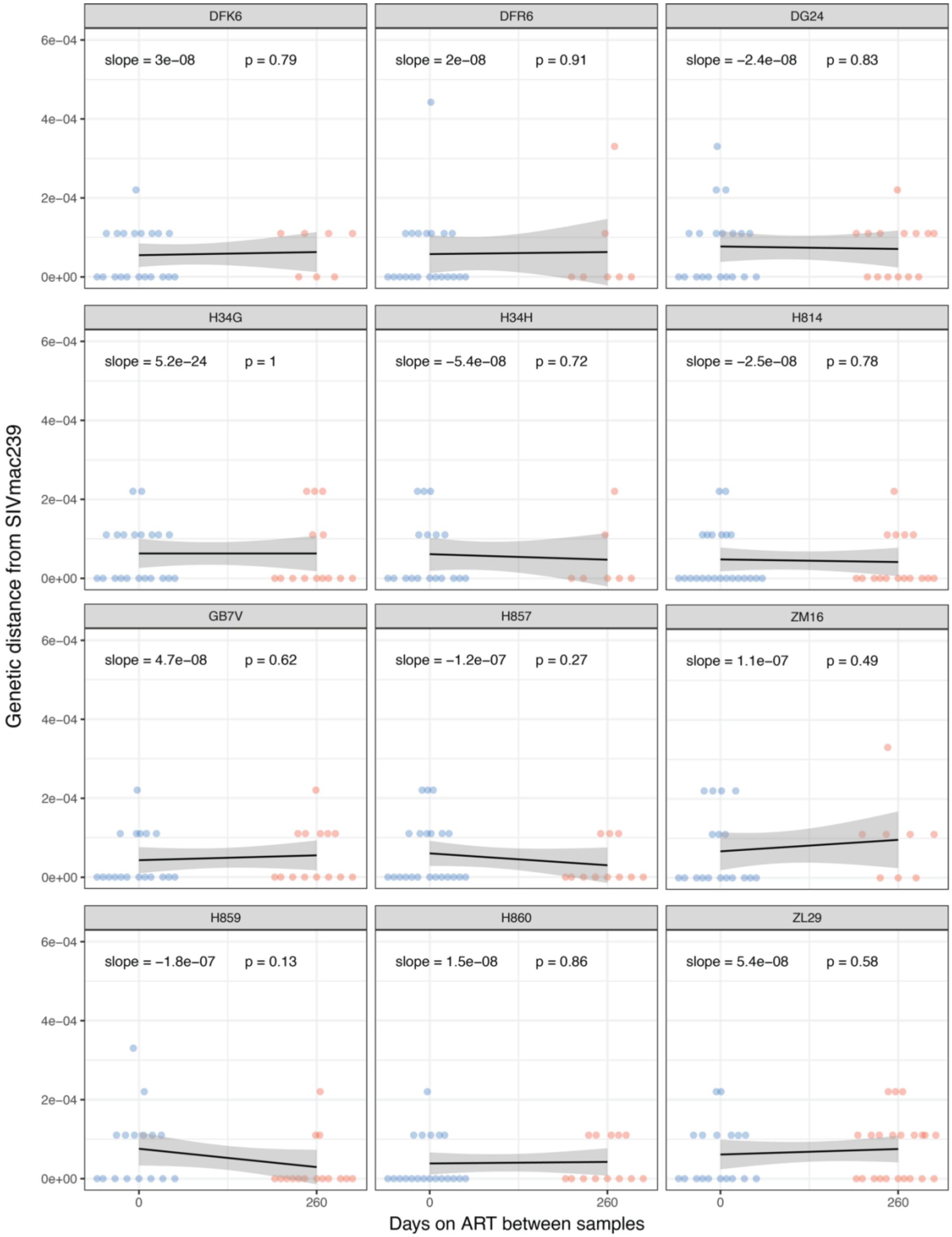

Supplemental Figure 3. Evolutionary rates for cohort 2 animals starting ART at 10 dpi. The scatter plot at each time point shows the distribution of p-distances from the SIVmac239 founder with black lines corresponding to the linear regression slopes and the grey bands to the 95% confidence intervals. The evolutionary rates during ART are not significantly different from zero in any animal (two-sided *t*-test). Source data are provided as a Source Data file.

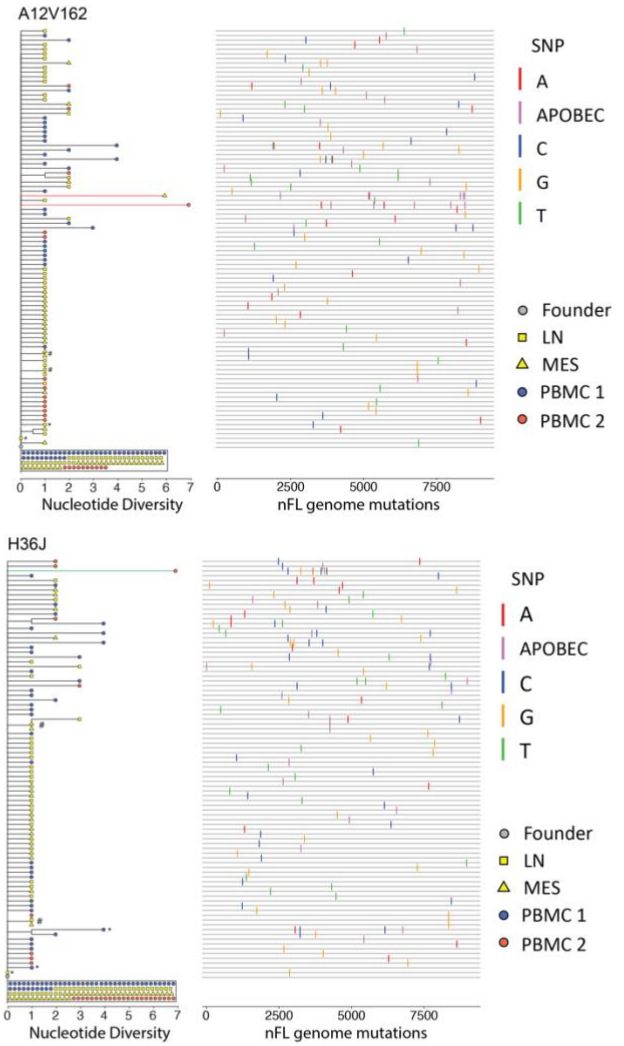

Supplemental Figure 4. Trees and highlighters for cohort 3 animals starting ART at 10 dpi. Phylogenetic trees for each individual RM are star-like with overall short branch lengths. Clades of potential viral clones (identical sequences with the same genetic barcode) are indicated by (#). Clades consisting of non-identical sequences with the same barcode are indicated by a diamond. Red lines indicate APOBEC-induced mutations and green indicate clustering of mutations. Single nucleotide polymorphisms (SNP) from the SIVmac239 founder sequence [red for adenine (A), blue for cytosine (C), orange for guanine (G) and green for thymine (T), including potential APOBEC-mediated mutations (purple)] are indicated in the highlighter plot.

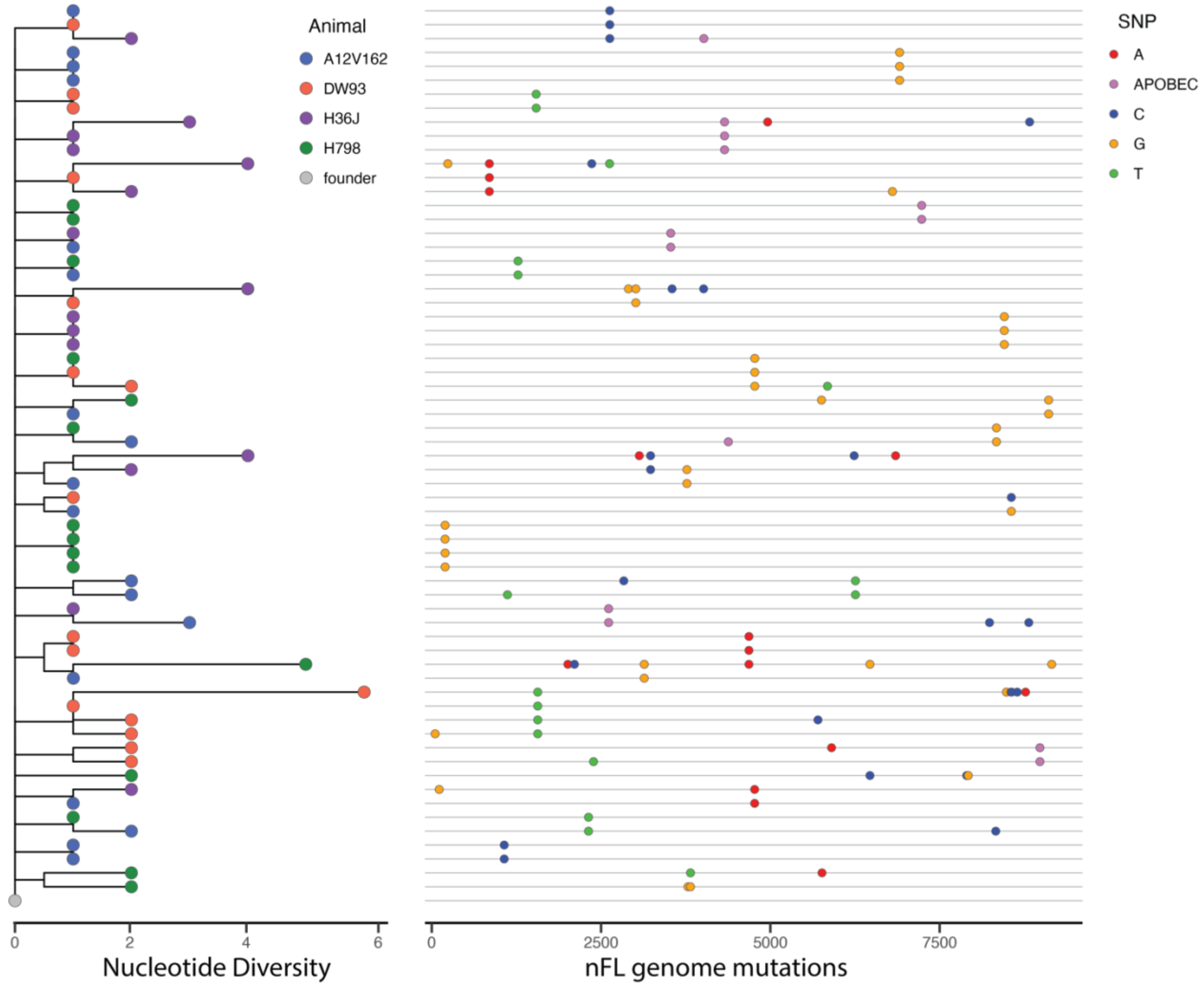

Supplemental Figure 5. Shared genetic mutations across all cohort 3 animals. A phylogenetic tree from containing only shared genetic mutations across all cohort 3 sequences is shown. The majority of clades consist of sequences from more than one animal. Sequences with indels are indicated with dots. Single nucleotide polymorphisms (SNP) from the SIVmac239 founder sequence [red for adenine (A), blue for cytosine (C), orange for guanine (G) and green for thymine (T), including potential APOBEC-mediated mutations (purple)] are indicated in the highlighter plot.

Supplemental Table 1. Evolutionary analyses with APOBEC-signature sites included. A two-sided Wilcoxon rank-sum test was used to assess changes in divergence and randomization tests were used to look for changes in APD and shifts in population structure. The evolutionary rate was estimated using linear regression, with the *t*-test used to assess if the slope was equal to zero. The p-values are not adjusted for multiple comparisons.

Group	Animal	Day	Tissue	Divergence	Diversity	Panmixia	Mutations site^{-1} day^{-1} (95% CI)	Pr(>|t|)	df
0	7811	17	PBMC	1.23E-03	**4.00E-03**[1]	**2.00E-03**	1.79e-05 (1e-05 - 2.57e-05)	**1.27E-04**	20
0	25611	14	PBMC	6.15E-04	7.73E-02	**1.08E-02**	1.81e-05 (7.49e-06 - 2.87e-05)	**1.53E-03**	30
1	DEVJ	345	PBMC	3.98E-01	2.64E-01	6.46E-01	-2.37e-07 (-6.38e-07 - 1.65e-07)	2.41E-01	40
1	DEVW	289	PBMC	2.96E-01	2.44E-01	6.96E-01	-1.57e-07 (-4.31e-07 - 1.18e-07)	2.55E-01	39
1	DEVX	351	PBMC	1.08E-01	2.28E-01	**3.34E-02**	-2.35e-07 (-5.87e-07 - 1.16e-07)	1.82E-01	32
1	H106	345	PBMC	3.67E-01	6.59E-01	1.74E-01	-1.24e-07 (-5.09e-07 - 2.61e-07)	5.17E-01	35
1	ZJ15	345	PBMC	6.40E-01	8.95E-01	7.70E-01	-2.74e-08 (-3.08e-07 - 2.53e-07)	8.44E-01	38
2	DFK6	260	PBMC	1.00E+00	9.94E-01	5.62E-01	-1.21e-08 (-2.36e-07 - 2.11e-07)	9.12E-01	25
2	DFR6	260	PBMC	6.27E-01	9.11E-01	5.59E-01	-2.06e-08 (-4.06e-07 - 3.65e-07)	9.13E-01	26
2	DG24	260	PBMC	5.83E-01	4.21E-01	9.73E-01	-7.87e-08 (-3.2e-07 - 1.63e-07)	5.12E-01	32
2	GB7V	260	PBMC	7.91E-01	9.96E-01	2.86E-01	2.35e-08 (-1.87e-07 - 2.34e-07)	8.21E-01	30
2	H34G	260	PBMC	9.55E-01	8.33E-01	8.73E-01	-4.03e-08 (-3.99e-07 - 3.19e-07)	8.21E-01	33
2	H34H	260	PBMC	9.46E-01	9.26E-01	4.78E-01	-1.68e-08 (-3.21e-07 - 2.88e-07)	9.10E-01	23
2	H814	260	PBMC	8.17E-01	7.42E-01	9.27E-01	-3.57e-08 (-2.29e-07 - 1.57e-07)	7.10E-01	37
2	H857	260	PBMC	9.21E-02	6.30E-02	5.83E-01	-2.11e-07 (-4.41e-07 - 1.82e-08)	6.98E-02	31
2	H859	260	PBMC	2.00E-01	2.61E-01	2.31E-01	-1.5e-07 (-3.87e-07 - 8.67e-08)	2.05E-01	29
2	H860	260	PBMC	3.13E-01	4.06E-01	3.04E-01	1.03e-07 (-1.02e-07 - 3.08e-07)	3.13E-01	31
2	ZL29	260	PBMC	4.59E-01	5.56E-01	3.66E-01	7.93e-08 (-1.44e-07 - 3.02e-07)	4.76E-01	38
2	ZM16	260	PBMC	4.62E-01	6.10E-01	2.40E-01	1.12e-07 (-2.2e-07 - 4.44e-07)	4.94E-01	24
2	All	260	PBMC	7.60E-01	6.08E-01	7.26E-01	-1.64e-08 (-8.73e-08 - 5.46e-08)	6.51E-01	381
3	A12V162	714	LN	7.73E-01	5.12E-01	4.59E-01	-9.85e-09 (-5.22e-08 - 3.25e-08)	6.46E-01	129
3	A12V162	714	MES	7.83E-01	9.93E-01	5.70E-01	5.04e-09 (-4.99e-08 - 5.99e-08)	8.56E-01	116
3	A12V162	714	ALL	7.39E-01	7.23E-01	5.26E-01	-3.28e-09 (-4.34e-08 - 3.68e-08)	8.72E-01	178
3	A12V162	1142	PBMC	1.24E-01	1.48E-01	6.27E-02	3.67e-08 (-1.25e-08 - 8.58e-08)	1.42E-01	91
3	A12V162	714+	All	4.70E-01	8.16E-01	5.74E-01	1.78e-08 (-1.87e-08 - 5.43e-08)	3.36E-01	202
3	DW93	714	LN	5.38E-01	9.25E-01	2.73E-01	3.39e-09 (-5.24e-08 - 5.91e-08)	9.04E-01	115
3	DW93	714	SPL	9.36E-01	9.75E-01	5.35E-01	7.97e-09 (-9.48e-08 - 1.11e-07)	8.78E-01	83
3	DW93	714	ALL	6.21E-01	9.17E-01	4.87E-01	4.32e-09 (-4.82e-08 - 5.68e-08)	8.71E-01	126
3	H36J	714	LN	3.56E-01	1.59E-01	3.67E-01	-3.41e-08 (-8.19e-08 - 1.38e-08)	1.61E-01	140
3	H36J	714	MES	4.02E-01	1.06E-01	3.54E-01	-3.72e-08 (-8.48e-08 - 1.03e-08)	1.24E-01	136
3	H36J	714	ALL	2.91E-01	5.16E-02	1.15E-01	-3.56e-08 (-7.29e-08 - 1.67e-09)	6.11E-02	197
3	H36J	1142	PBMC	2.88E-01	8.17E-01	6.71E-01	-5.23e-09 (-5.17e-08 - 4.12e-08)	8.24E-01	111
3	H36J	714+	All	2.20E-01	1.33E-01	8.95E-02	-1.79e-08 (-5.09e-08 - 1.52e-08)	2.88E-01	229
3	H798	714	LN	4.98E-01	6.42E-01	9.47E-01	-1.06e-08 (-5.56e-08 - 3.44e-08)	6.42E-01	113
3	H798	714	MES	6.12E-02	**2.39E-02**	**2.03E-02**	7.26e-08 (6.98e-09 - 1.38e-07)	**3.04E-02**	107
3	H798	714	SPL	2.87E-01	2.43E-01	7.51E-01	-2.63e-08 (-7.47e-08 - 2.2e-08)	2.82E-01	99
3	H798	714	ALL	9.00E-01	5.69E-01	9.90E-01	1.34e-08 (-3.1e-08 - 5.77e-08)	5.53E-01	183
3	H798	1142	PBMC	5.85E-01	4.63E-01	2.77E-01	1.42e-08 (-2.24e-08 - 5.08e-08)	4.43E-01	95
3	H798	714+	All	7.88E-01	4.83E-01	1	1.38e-08 (-2.22e-08 - 4.99e-08)	4.50E-01	210

[1] P-values < 0.05 are highlighted in bold.
